# Titanium-Prepared Versus Leukocyte Platelet-Rich Fibrin With Demineralized Freeze-Dried Bone Allograft in Periodontal Intrabony Defects: A Prospective Observational Study

**DOI:** 10.7759/cureus.106347

**Published:** 2026-04-02

**Authors:** Sohini Chakraborty, Ellora Madan, Purushottam Kumar, Tanusmitha Pathak, Aparna Mishra, Narayani Singh, Nuzla Agha, Karnika Agrawal, Seema Gupta

**Affiliations:** 1 Department of Periodontology, Kothiwal Dental College and Research Centre, Moradabad, IND; 2 Department of Oral Medicine and Radiology, Kothiwal Dental College and Research Centre, Moradabad, IND; 3 Department of Orthodontics, Kothiwal Dental College and Research Centre, Moradabad, IND

**Keywords:** bone regeneration, periodontal attachment loss, periodontal disease, periodontitis, platelet-rich fibrin

## Abstract

Introduction: Periodontal intrabony defects represent a major challenge in regenerative therapy, owing to the significant loss of supporting bone and attachment. Platelet-rich fibrin (PRF) variants, including leukocyte-PRF (L-PRF) and titanium-prepared PRF (T-PRF), are autologous second-generation concentrates that enhance healing through the sustained release of growth factors, while demineralized freeze-dried bone allograft (DFDBA) provides an osteoinductive and osteoconductive scaffold. The aim of the present study was to compare the clinical and radiographic efficacy of T-PRF + DFDBA versus L-PRF + DFDBA in the regenerative treatment of periodontal intrabony defects over six months using cone-beam computed tomography (CBCT) and clinical parameters.

Methods: This prospective observational study included 80 intrabony defect sites in systemically healthy patients (40 sites per group). The sites were assigned to Group A (T-PRF + DFDBA) or Group B (L-PRF + DFDBA) based on clinical judgment and feasibility. The probing pocket depth (PPD), clinical attachment level (CAL), and early healing index were recorded at baseline, three months, and six months. Intrabony defect depth, crestal level, and bone fill were assessed via CBCT at baseline and six months. Data were analyzed using repeated-measures analysis of variance (ANOVA), paired t-test, and independent t-test.

Results: Both groups showed highly significant intragroup improvements in PPD, CAL, intrabony defect depth, crestal level, and bone fill over six months (p < 0.001). Intergroup comparisons revealed no statistically significant differences in PPD (baseline: 7.82 ± 0.90 mm vs 7.77 ± 0.86 mm; 6 months: 2.86 ± 0.56 mm vs 2.95 ± 1.05 mm), CAL, early healing score (1.36 ± 0.49 vs 1.23 ± 0.42; p = 0.327), bone fill (1.73 ± 1.35 mm vs 1.75 ± 1.07 mm; p = 0.961), crestal level gain, or intrabony defect reduction at any time point (all p > 0.05).

Conclusions: T-PRF + DFDBA and L-PRF + DFDBA provided comparable and clinically meaningful regenerative outcomes in periodontal intrabony defects over six months, with no evidence of superiority of one approach over the other. The dominant role of DFDBA likely accounts for the equivalence observed, supporting the interchangeable clinical use of either PRF variant as an adjunct to DFDBA for routine periodontal regeneration.

## Introduction

Chronic periodontitis is a prevalent inflammatory condition characterized by progressive destruction of periodontal supporting tissues, including bone resorption, loss of clinical attachment, and formation of periodontal pockets [1]. Among the various osseous defects associated with this disease, intrabony defects (also known as vertical or angular defects) represent a significant challenge because they compromise tooth stability and increase the risk of further periodontal breakdown and eventual tooth loss [2]. Effective management of these defects aims not only to halt disease progression but also to achieve true periodontal regeneration, including restoration of lost bone, periodontal ligament, and cementum.

Conventional non-regenerative approaches, such as scaling and root planing combined with open-flap debridement, often result in repair rather than regeneration with limited bone fill and clinical attachment gain [3]. To overcome these limitations, regenerative strategies that incorporate bone graft materials and bioactive agents have been developed. Demineralized freeze-dried bone allograft (DFDBA) has emerged as a widely used xenogeneic/allogeneic graft material because of its osteoinductive properties, which are attributed to the presence of bone morphogenetic proteins (BMPs) such as BMP-2, -4, and -7. DFDBA provides a scaffold for new bone formation and has demonstrated superior outcomes in intrabony defect resolution compared to debridement alone [4,5].

The advent of second-generation platelet concentrates, particularly platelet-rich fibrin (PRF), has further advanced regenerative periodontal therapy [6]. Leukocyte-platelet-rich fibrin (L-PRF) is prepared by centrifuging venous blood without anticoagulants in glass tubes, yielding a dense fibrin matrix rich in platelets, leukocytes, and growth factors that are released gradually over 7-14 days. This promotes angiogenesis, cell migration, proliferation, and tissue healing [7]. To enhance biocompatibility and fibrin quality, titanium-prepared PRF (T-PRF) was proposed by Tunali et al., who utilized titanium tubes to stimulate stronger platelet activation and a more structured, slower-resorbing fibrin network with potentially improved mechanical properties and biological activity [8].

Although both L-PRF and T-PRF have shown promise in periodontal applications, limited comparative evidence exists regarding their efficacy when combined with DFDBA for treating intrabony defects, particularly when using advanced imaging such as cone-beam computed tomography (CBCT) for precise assessment of bone fill and crestal level changes [9].

The aim of the present study was to compare the efficacy of T-PRF and L-PRF membranes, each in conjunction with DFDBA, in the regenerative treatment of periodontal intrabony defects, evaluated using clinical and CBCT-based radiographic parameters over a six-month period. The specific objectives included assessing intragroup and intergroup differences in probing pocket depth (PPD), clinical attachment level (CAL), early healing index, intrabony defect reduction, postoperative bone fill, and crestal level changes.

## Materials and methods

This study was designed as a prospective observational cohort study without randomization or interventional assignment by the investigators, allowing for natural group allocation based on clinical judgment, patient-specific factors, operator preference, and treatment feasibility in routine clinical practice. The study was carried out in the Department of Periodontology at Kothiwal Dental College and Research Centre, Moradabad, India, from July 2023 to March 2025, with each enrolled participant followed prospectively for six months post-surgery. The study protocol was approved by the Institutional Ethical Review Board of the institution (KDCRC/IERB/04/2023/11 dated 07-04-23) and adhered to the ethical principles outlined in the Declaration of Helsinki. Written informed consent was obtained from all participants after providing a detailed explanation of the study procedures, potential benefits, risks, alternative treatments, and the voluntary nature of participation, including the right to withdraw at any time without affecting their care.

The eligibility criteria included adult patients (aged 18-60 years) diagnosed with Stage III periodontitis (grades A-C) presenting with at least one periodontal intrabony defect, characterized by PPD ≥5 mm and CAL ≥3 mm (with interdental CAL ≥5 mm at sites of greatest loss), gingival index ≥1, and radiographically confirmed intrabony defect depth ≥3 mm on CBCT [10]. Exclusion criteria included systemic diseases affecting periodontal healing (such as uncontrolled diabetes and autoimmune disorders), pregnancy or lactation, bleeding disorders, history of periodontal therapy within the previous year, smoking >10 cigarettes per day, and use of medications known to impair gingival healing (such as long-term corticosteroids and immunosuppressants).

The sample size was calculated using G*Power software (Heinrich-Heine-Universität Düsseldorf, Düsseldorf, Germany) based on an effect size of 0.56 from a prior study, with 80% power and alpha error of 0.05, yielding a minimum of 36 participants per group [11]. Accounting for an anticipated 10% loss to follow-up, the sample size was increased to 40 sites per group.

Eligible sites were prospectively assigned to one of two observational groups: Group A received a T-PRF membrane combined with DFDBA, and Group B received an L-PRF membrane combined with DFDBA. Group allocation was non-random and determined by clinical indications such as defect morphology, patient preference, or procedural practicality, reflecting real-world application rather than experimental randomization. The pre-surgical phase involved detailed medical and dental history recording, followed by full-mouth scaling and root planing using ultrasonic and hand instruments, oral hygiene instructions emphasizing proper brushing techniques, and re-evaluation after three to four weeks to confirm baseline parameters and plaque control.

Surgical procedures were performed under local anesthesia using 2% lignocaine with 1:80,000 adrenaline (Lignox 2% A, Neon Laboratories Ltd., Mumbai, India). A full-thickness mucoperiosteal flap was reflected, intrabony defects were thoroughly debrided, and root surfaces were planed with Gracey curettes to achieve a smooth, hard surface. DFDBA (Dembone, Pacific Coast Tissue Bank, Los Angeles, California, USA) was hydrated with the patient's blood and placed into the defect as a scaffold. For Group A, T-PRF was prepared by collecting 5 ml of venous blood in a sterile titanium tube without anticoagulant, followed by immediate centrifugation at 2800 rpm for 12 min using a laboratory centrifuge (Remi R-8C, Remi Elektrotechnik Limited, Vasai, India), which was then separated and gently compressed into a membrane [9].

For Group B, L-PRF was prepared similarly by collecting 5 ml of blood without an anticoagulant and centrifuging at 2700 rpm for 12 min, with the fibrin clot compressed into a membrane [11]. The respective PRF membrane was placed over the grafted site, followed by flap repositioning, suturing with 3-0 black silk sutures, and application of periodontal dressing. Postoperative care included 0.2% chlorhexidine mouthwash (Hexidine, ICPA Health Products Ltd., Mumbai, India) for one week, avoidance of mechanical oral hygiene at the surgical site initially, analgesics, and antibiotics, as indicated, and regular follow-up visits for maintenance.

The primary clinical outcomes included PPD and CAL measured at baseline, three months, and six months using a UNC-15 periodontal probe with a custom acrylic stent to improve reproducibility. Radiographic outcomes included intrabony defect depth (IBD), crestal level (CL), and bone fill assessed using CBCT scans (Carestream Dental CS 9300, Atlanta, Georgia, US) at baseline and six months, with standardized imaging protocols to ensure comparability. All measurements were performed by a single calibrated examiner to minimize variability, with the same operator conducting all the surgeries.

Centrifugation parameters were further standardized by reporting the relative centrifugal force (RCF). Based on the centrifuge rotor radius (approximately 8 cm), the applied protocols corresponded to approximately 708 ×g at 2700 rpm and 760 ×g at 2800 rpm. Cone-beam computed tomography acquisition was performed using standardized settings, including a voxel size of 0.18 mm, a field of view of 5 × 5 cm, tube voltage of 90 kVp, tube current of 10 mA, and exposure time of 12 seconds. Examiner calibration was performed prior to the study by repeating measurements at 10 randomly selected sites at a one-week interval. Intra-examiner reliability was assessed using the intraclass correlation coefficient, which demonstrated excellent agreement (ICC = 0.91-0.95).

Data were analyzed using IBM SPSS Statistics software (version 26.0; IBM Corp., Armonk, New York, US). Normality was assessed using the Shapiro-Wilk test. Descriptive statistics included the mean ± standard deviation for continuous variables and frequencies/percentages for categorical variables. Intragroup comparisons over time were performed using repeated-measures analysis of variance (ANOVA) with Bonferroni post-hoc tests for periodontal parameters and paired t-tests for radiographic changes. Intergroup comparisons were performed using independent t-tests (for normally distributed data). Statistical significance was set at P < 0.05.

## Results

This prospective observational study included 80 periodontal intrabony defect sites (40 per Group) treated with either titanium-prepared platelet-rich fibrin + demineralized freeze-dried bone allograft (group A) or leukocyte-platelet-rich fibrin + demineralized freeze-dried bone allograft (Group B). The groups were comparable at baseline in terms of demographic characteristics (age and sex) and the initial clinical and radiographic parameters (Table 1).

**Table 1:** Demographic details of study groups Continuous data such as age are presented as mean and standard deviation, whereas categorical data such as sex are presented as frequency (n) and percentage (%), p > 0.05 denotes no statistical significance using independent t-test for age and chi-square test for sex, Titanium-prepared platelet-rich fibrin + demineralized freeze-dried bone allograft (Group A) and leukocyte-platelet rich fibrin + demineralized freeze-dried bone allograft (Group B).

Parameters	Unit	Group A (n = 40)	Group B (n = 40)	Test value	p-value
Age	Years, Mean ± SD	34.56 ± 8.23	32.82 ± 6.45	t = 1.055	0.295
Sex	Male, n%	24 (60%)	18 (45%)	χ² = 1.805	0.179
	Female, n%	16 (40%)	22 (55%)		

Both treatment modalities resulted in significant intragroup improvements in the clinical parameters over the six-month period (Table 2). PPD and CAL showed progressive and statistically significant reductions at three and six months in both groups (p = 0.001 for all intragroup comparisons).

**Table 2:** Intragroup comparison of periodontal parameters at different time intervals (using repeated ANOVA test). *Significant at p < 0.05 (repeated-measures analysis of variance), are presented as mean and standard deviation, Titanium-prepared platelet-rich fibrin + demineralized freeze-dried bone allograft (Group A) and leukocyte-platelet rich fibrin + demineralized freeze-dried bone allograft (Group B).

Parameter	Groups	Baseline ( Mean ± SD)	3 months ( Mean ± SD)	6 months ( Mean ± SD)	F value	p-value
Probing pocket depth (mm)	Group A	7.82 ± 0.90	3.95 ± 0.65	2.86 ± 0.56	306.16	0.001*
	Group B	7.77 ± 0.86	4.36 ± 0.90	2.95 ± 1.05	568.79	0.001*
Clinical attachment level (mm)	Group A	11.23 ± 1.77	7.55 ± 2.15	7.82 ± 2.19	90.82	0.001*
	Group B	11.55 ± 1.81	7.09 ± 1.82	7.14 ± 1.72	276.14	0.001*

Post-hoc analysis confirmed significant changes between all time points for PPD and between baseline and follow-up visits for CAL, with CAL gains stabilizing after three months (Table 3).

**Table 3:** Post-hoc analysis of periodontal parameters using Bonferroni test *Significant at p < 0.05 (Bonferroni post-hoc test), Titanium-prepared platelet-rich fibrin + demineralized freeze-dried bone allograft (Group A) and leukocyte-platelet rich fibrin + demineralized freeze-dried bone allograft (Group B).

Group	Paired time point	Probing pocket depth		Clinical attachment level	
		t-value	p-value	t-value	p-value
Group A	Baseline vs. 3 months	24.68	< 0.001*	9.42	< 0.001*
	Baseline vs. 6 months	31.42	< 0.001*	8.76	< 0.001*
	3 months vs. 6 months	8.94	< 0.001*	-1.24	0.223
Group B	Baseline vs. 3 months	18.92	< 0.001*	14.28	< 0.001*
	Baseline vs. 6 months	22.76	< 0.001*	13.95	< 0.001*
	3 months vs. 6 months	7.85	< 0.001*	-0.31	0.758

Radiographic assessment using cone-beam computed tomography demonstrated significant intragroup improvements at six months in both groups (Table 4), including a reduction in intrabony defect depth and gain at the crestal level (p = 0.001 for all comparisons).

**Table 4:** Intragroup comparison of alveolar bone parameters (using paired t test) at different time intervals. *Significant at p < 0.05 (paired t-test), Titanium-prepared platelet-rich fibrin + demineralized freeze-dried bone allograft (Group A) and leukocyte-platelet rich fibrin + demineralized freeze-dried bone allograft (Group B).

Parameter	Groups	Baseline ( Mean ± SD)	6 months ( Mean ± SD)	Mean Difference	t value	p-value
Intrabony defect (mm)	Group A	11.31 ± 2.56	9.57 ± 2.78	1.74	6.02	0.001*
	Group B	11.43 ± 2.47	9.68 ± 2.43	1.75	7.64	0.001*
Crestal level (mm)	Group A	10.05 ± 2.29	8.27 ± 2.62	1.78	7.98	0.001*
	Group B	10.18 ± 2.14	8.68 ± 2.28	1.51	6.33	0.001*

Intergroup comparisons revealed no statistically significant differences between groups A and B for PPD or CAL at baseline, three months, or six months (Table 5; all p > 0.05). Similarly, no significant intergroup differences were observed in crestal level, intrabony defect depth at baseline and six months (Table 6; all p > 0.05), or postoperative bone fill at six months (Table 7; p = 0.961).

**Table 5:** Comparison of periodontal parameters between groups (using independent t test) at multiple time intervals No significant intergroup differences at any time point (p > 0.05), Titanium-prepared platelet-rich fibrin + demineralized freeze-dried bone allograft (Group A) and leukocyte-platelet rich fibrin + demineralized freeze-dried bone allograft (Group B).

Groups	Baseline (Mean ± SD)	t value	p-value	3 months ( Mean ± SD)	t value	p-value	6 months ( Mean ± SD)	t value	p-value
Probing pocket depth (mm)									
Group A	7.82 ± 0.90	0.17	0.866	3.95 ± 0.65	1.72	0.092	2.86 ± 0.56	0.36	0.721
Group B	7.77 ± 0.86			4.36 ± 0.90			2.95 ± 1.05		
Clinical attachment level (mm)									
Group A	11.23 ± 1.77	0.59	0.56	7.55 ± 2.15	0.76	0.454	7.82 ± 2.19	1.14	0.259
Group B	11.55 ± 1.81			7.09 ± 1.82			7.14 ± 1.72		

**Table 6:** Comparison of alveolar bone parameters between groups (using independent t test) at multiple time intervals No significant difference between groups (p > 0.05) using independent t-test, Titanium-prepared platelet-rich fibrin + demineralized freeze-dried bone allograft (Group A) and leukocyte-platelet rich fibrin + demineralized freeze-dried bone allograft (Group B).

Groups	Baseline ( Mean ± SD)	t value	p-value		6 months ( Mean ± SD)	t value	p-value
Crestal level (mm)							
Group A	10.05 ± 2.29	0.20	0.841		8.27 ± 2.62	0.55	0.584
Group A	10.18 ± 2.14				8.68 ± 2.28		
Intrabony defect (mm)							
Group A	11.31 ± 2.56	0.16	0.873	9.57 ± 2.78		0.13	0.895
Group B	11.43 ± 2.47			9.68 ± 2.43			

**Table 7:** Intergroup comparison of postoperative bone fill (in mm) at six months (using independent t test) No significant difference between groups (p > 0.05) using independent t-test, Titanium-prepared platelet-rich fibrin + demineralized freeze-dried bone allograft (Group A) and leukocyte-platelet rich fibrin + demineralized freeze-dried bone allograft (Group B).

Groups	6 months ( Mean ± SD)	Mean Difference	t value	p-value
Group A	1.73 ± 1.35	0.02	0.05	0.961
Group B	1.75 ± 1.07			

## Discussion

The findings of this prospective observational study indicate that both T-PRF combined with DFDBA and L-PRF combined with DFDBA produced comparable regenerative outcomes in periodontal intrabony defects over six months. The lack of statistically significant intergroup differences in PPD, CAL, intrabony defect resolution, crestal level gain, postoperative bone fill, or early wound healing suggests that the two PRF variants, when used as adjuncts to DFDBA, offer equivalent clinical utility in routine periodontal practice.

Several biological and structural differences between T-PRF and L-PRF could theoretically lead to divergent performances, yet these did not translate into detectable superiority in the present cohort. L-PRF contains a higher concentration of leukocytes, which contribute additional pro-inflammatory and antimicrobial cytokines along with a denser, more stable fibrin architecture that supports prolonged growth factor release (platelet-derived growth factor, transforming growth factor-beta, and vascular endothelial growth factor) [12]. This matrix is thought to favor soft tissue healing and early epithelial coverage, which may explain the trend toward a slightly greater PPD reduction observed with L-PRF.

In contrast, T-PRF prepared in titanium tubes exhibits enhanced platelet activation, improved fibrin tensile strength, and a slower resorption profile, potentially providing better mechanical protection and sustained osteogenic signaling in the crestal zone, consistent with the modest numerical advantage in crestal level gain seen in that group [8,13]. The absence of significant intergroup differences implies that these subtle compositional variations are largely neutralized when DFDBA is used as the primary scaffold, whose osteoinductive bone morphogenetic proteins and osteoconductive surfaces dominate the regeneration process.

These results are broadly consistent with those of earlier investigations that combined PRF variants with bone grafts for intrabony defects. MItra et al. conducted a randomized clinical trial comparing T-PRF and L-PRF in the treatment of intrabony defects without additional bone grafts [14]. They found significant intragroup improvements in clinical and radiographic parameters in both groups; however, intergroup analysis showed that T-PRF achieved superior defect fill compared with L-PRF, concluding that T-PRF may serve as a better alternative because of its enhanced biocompatibility and structural advantages. Similarly, Oza et al. conducted a systematic review and meta-analysis to evaluate the clinical efficacy of T-PRF in periodontal regeneration, and concluded that T-PRF demonstrated favorable outcomes in promoting bone fill, CAL gain, and PPD reduction, with enhanced biocompatibility and structural advantages over conventional PRF in various regenerative applications [15]. The results of our study differed primarily because DFDBA was used as the main regenerative scaffold in both groups, likely due to the subtle differences between T-PRF and L-PRF.

The equivalence observed in our study, where both T-PRF + DFDBA and L-PRF + DFDBA yielded similar regenerative outcomes without significant intergroup differences, is strongly supported by the findings of Haripriya et al. [9] and Alshoiby et al. [16]. These randomized trials demonstrated that DFDBA alone produced comparable clinical attachment gain, probing pocket depth reduction, and radiographic bone fill as DFDBA augmented with PRF variants (T-PRF or injectable PRF), indicating no added benefit from the PRF component. This reinforces that the primary osteoinductive and osteoconductive properties of DFDBA likely dominate the regeneration process in intrabony defects, overshadowing any subtle biological advantages of one PRF preparation over the other and explaining the lack of superiority between T-PRF and L-PRF in the observational cohort.

Theodosaki et al. conducted a systematic review and meta-analysis evaluating the effectiveness of PRF alone or combined with bone grafts in treating periodontal infrabony defects and concluded that PRF provided statistically significant benefits in CAL, PPD, and bone fill at six months follow-up, particularly when used adjunctively, although differences diminished by nine months [17]. Ardila et al. conducted an umbrella review synthesizing high-level evidence on the clinical efficacy of platelet derivatives (including PRF variants) in periodontal tissue regeneration and concluded that therapies using platelet derivatives in intrabony defects generally provided superior regenerative outcomes compared with monotherapies, although the results varied by specific application and combination with grafts [18]. This comprehensive synthesis supports the adjunctive benefits of PRF in periodontal regeneration while highlighting contexts where differences among platelet preparations may be minimal when combined with bone substitutes.

The comparable early healing scores further support the notion that both preparations facilitate rapid initial wound stabilization, likely due to the shared ability of PRF to create a biologically active seal that protects the graft and promotes hemostasis. In the context of an observational design, the lack of randomization may have introduced selection bias; however, baseline comparability across key variables minimizes this concern and enhances the generalizability of the findings to real-world clinical settings, where treatment choice is guided by defect morphology, patient factors, and operator experience rather than strict allocation protocols.

Clinical implications

The equivalence of T-PRF and L-PRF when combined with DFDBA implies that clinicians may select either preparation based on availability, cost, ease of preparation, or personal preference, without compromising expected regenerative outcomes. Both approaches appear to offer predictable improvements in pocket closure, attachment gain, and radiographic bone fill, making them valuable adjuncts for the management of moderate-to-deep intrabony defects. The choice between the two may be influenced by case-specific needs (such as prioritizing soft tissue healing in esthetic zones or crestal stability in non-contained defects).

Limitations

The observational nature of the study precludes causal inference and increases the susceptibility to selection and confounding bias. The six-month follow-up period provided insight into the long-term stability of the regenerated tissues. The absence of histological evaluation prevented the confirmation of true periodontal regeneration versus repair. The sample, while adequately powered for the chosen effect size, remains relatively modest, potentially underdetecting small but clinically relevant differences. Finally, the single-center design and lack of blinding may introduce operator or measurement bias, although standardized protocols and a single examiner were employed to mitigate these issues. Future randomized controlled trials with longer observation periods, histological analyses, and larger multicenter samples are warranted to confirm these observations and explore potential subgroup effects.

An additional limitation of this study is the potential for allocation bias due to clinician-driven group assignment. Although baseline clinical and radiographic parameters were comparable between groups, unmeasured confounders such as defect morphology (e.g., number of bony walls), tooth position, and patient-related factors may have influenced treatment outcomes. Furthermore, no stratification or multivariable analysis was performed to adjust for these covariates. Future randomized controlled trials with adjusted analyses are recommended to minimize such bias and better isolate treatment effects.

## Conclusions

This prospective observational study demonstrated that titanium-prepared platelet-rich fibrin combined with demineralized freeze-dried bone allograft and leukocyte platelet-rich fibrin combined with demineralized freeze-dried bone allograft are equally effective in the regenerative treatment of periodontal intrabony defects over a six-month period. Both approaches yielded comparable improvements in probing pocket depth reduction, clinical attachment gain, early wound healing, and radiographic parameters, including intrabony defect resolution, crestal level improvement, and bone fill, with no statistically significant differences between the groups. These findings suggest that the dominant regenerative contribution of DFDBA as an osteoinductive and osteoconductive scaffold likely minimizes subtle biological distinctions between the two PRF variants. Clinically, these results support the interchangeable use of T-PRF or L-PRF as adjuncts to DFDBA in routine periodontal practice, offering predictable regeneration without evidence of the superiority of one preparation over the other.
